# Assessing health status in informal schizophrenia caregivers compared with health status in non-caregivers and caregivers of other conditions

**DOI:** 10.1186/s12888-015-0547-1

**Published:** 2015-07-21

**Authors:** Shaloo Gupta, Gina Isherwood, Kevin Jones, Kristel Van Impe

**Affiliations:** 1 Health Outcomes Practice, Kantar Health, 1 Independence Way, Suite 220, Princeton, NJ 08540 USA; 2 Kantar Health, Epsom, Surrey UK; 3 European Federation of Associations of Families of People with Mental Illness, Diestsevest 100, B-3000 Leuven, Belgium; 4 Janssen-Cilag GmbH, Neuss, Germany

**Keywords:** Caregivers, Schizophrenia, Health-Related Quality of Life, Comorbidities, Health Utilities, Depression

## Abstract

**Background:**

Research indicates schizophrenia is a cause of burden for patients and caregivers. This study examined health-related quality of life (HRQoL) and comorbidities experienced by informal schizophrenia caregivers compared with non-caregivers and caregivers of adults with other conditions (*e.g.*, Alzheimer’s disease, cancer, and stroke).

**Methods:**

Data were obtained from the 5EU (France, Germany, Italy, Spain, UK) National Health and Wellness Survey, an online questionnaire that is representative of the total 5EU adult (18+ years) population. Respondents provided information on HRQoL (SF-36v2: mental and physical component summary (MCS, PCS) and SF-6D (health utility) scores), items from the Caregiver Reaction Assessment (strongly disagree to strongly agree) and comorbidities (sleep difficulties, insomnia, pain, headaches, heartburn, anxiety, depression) experienced in the past 12 months. Schizophrenia caregivers (*n* = 398) were matched to non-caregivers (*n* = 158,989) and caregivers of other conditions (*n* = 14,341) on baseline characteristics *via* propensity scores. Chi-square tests and ANOVAs were used to determine significant differences across groups.

**Results:**

The average age of schizophrenia caregivers was 45.3 years (SD = 15.8), and 59.6 % were female. After matching, schizophrenia caregivers reported lower MCS (40.3 *vs.* 45.9), PCS (46.8 *vs.* 49.0), and health utilities (0.64 *vs.* 0.71) compared with non-caregivers (all *p* < 0.001). Schizophrenia caregivers were more likely to experience sleep difficulties (42.7 % *vs.* 28.5 %), insomnia (32.4 % *vs.* 18.5 %), pain (39.7 % *vs.* 30.4 %), headaches (48.0 % *vs.* 42.0 %), heartburn (31.7 % *vs.* 22.9 %), anxiety (37.9 % *vs.* 23.6 %), and depression (29.4 % *vs.* 19.4 %) than non-caregivers. Comparing schizophrenia caregivers and other caregivers, schizophrenia caregivers reported lower MCS (40.3 *vs.* 42.7, *p* < 0.001), and health utilities (0.64 *vs.* 0.67, *p* < 0.001). Schizophrenia caregivers were more likely to experience sleep difficulties, insomnia, pain, and anxiety than other caregivers. Almost 60 % of schizophrenia caregivers agree/strongly agree that caring for the patient is important to them, but only 31.9 % agree/strongly agree that they have the financial resources to provide adequate care.

**Conclusions:**

Schizophrenia caregivers reported worse HRQoL than non-caregivers and caregivers of other conditions. Providing care for an adult relative with schizophrenia is important to caregivers, but caregivers need more resources to provide adequate care. Providing informal schizophrenia caregivers with support services to help better manage patients may improve their health status.

## Background

Schizophrenia is a mental disorder that affects the patient’s ability to engage in day-to-day activities, which increases the patients’ reliance on caregivers to assist them. Objective caregiving burden involves the effects on the household such as assisting patients in daily tasks, which may include disrupted caregiver behavior and daily routine, among others [1, 2]. Subjective caregiving burden involves how caregivers perceive the burden of care, which may include shame, embarrassment, feelings of guilt, or self-blame, among others. Together, these have consequences on the physical, psychological, economic, and emotional well-being of schizophrenia caregivers [1, 3–6].

For schizophrenia caregivers, burden is intensified by the patient’s severity of symptoms, duration of illness, number of needs, disability extent, decreased social interests, being male, and being older [1–3, 7, 8]. In particular, schizophrenia with prominent positive or negative symptoms has been associated with lower functionality, health-related quality of life (HRQoL), and lost work days among caregivers, with greatest caregiver burden among patients with the highest symptomatology [9]. While no previous research, to the authors’ knowledge, have assessed whether pharmacological interventions which helps improve patients’ disease control are directly associated with decreased caregiver burden in schizophrenia, high-quality meta-analyses have shown that pharmacological interventions may have modest efficacy in mitigating burden and distress among caregivers. Indeed, many studies have shown improvements in caregiving burden associated symptoms, even when caregiver responsibilities were not alleviated [10].

It has been found that certain domains of caregiving burden, such as tension and stress, are associated with maladaptive coping mechanisms and increased risk of psychological morbidity in schizophrenia caregivers [11]. Yet, the “burden of care” is a complex construct often defined by the impact on caregivers, and often criticized for focusing only on the negative aspects of caregiving [1]. Notably, other domains of caregiving, such as satisfaction and meaning derived from caregiving, have been found to be negatively associated with subjective burden, and positively associated with HRQoL, among schizophrenia caregivers, elucidating the positive effects of caregiving. Indeed, the literature does suggest caregivers experience gains from their caregiving experience by becoming more sensitive to persons with disabilities, finding clarity in their priorities in life, and a greater sense of inner strength. Moreover, they experience positive personal experiences and positive aspects in their relationships with the patients. In turn, the positive aspects of caregiving have been associated with higher HRQoL among caregivers [12, 13].

Indeed, the World Health Organization (WHO) European Mental Health Plan guidelines advise that “The coping capacity and skills of families should be assessed regularly, and measures taken to ensure that families benefit from the necessary support, education and the provision of resources…” and that it is up to the public health system of each country to help “identify and provide resources to support families that look after loved ones requiring long-term care, including education, relief services and adequate benefits” [14].

While much research has been done to characterize the burden of providing care to schizophrenia patients, only a handful of studies have directly compared HRQoL and comorbidities for schizophrenia caregivers to non-caregiver controls and other caregivers. Moreover, previous studies have utilized non-representative and relatively small samples [4, 6]. To the authors’ knowledge, only two studies have directly compared caregiver burden in schizophrenia and caregivers of other conditions in Europe (France and Cyprus) [15, 16]. Although caring for both schizophrenia and patients with other conditions impacts the well-being of caregivers, differences in the onset of disease and disease symptoms may suggest that these diseases will affect different aspects of caregiver burden.

There were three primary purposes to the present study. First, given that the evidence on the health status of caregivers of adults with schizophrenia in the existing literature is limited in Europe, informal schizophrenia caregivers and non-caregiver controls across 5EU were compared on measures of HRQoL, and comorbidities experienced in the past 12 months. Second, informal schizophrenia caregivers were compared with adult caregivers of other conditions (*e.g.*, Alzheimer’s disease, cancer, stroke, etc.) on these same measures. Third, schizophrenia caregivers’ subjective assessment of the caregiving experience, both as a positive experience and as a burden, was characterized. This study uses the National Health and Wellness Survey (NHWS), which provides a large, nationally representative sample.

## Method

### Sample and procedure

The current study includes data from the 5EU NHWS. The NHWS is an annual, cross-sectional, self-administered questionnaire from a sample of adults aged 18 or older (www.kantarhealth.com). The following study included the following five EU countries: France, Germany, Italy, Spain, and United Kingdom (UK). The NHWS is intended to represent the entire adult population of that country by employing a stratified random sampling framework. The demographic distributions of each country are obtained from the International Database from the United States Bureau of the Census; potential respondents are selected in such a way as to mirror these characteristics. Specifically, the age and gender distributions are matched in the 5EU countries. Potential respondents are recruited from various online panels by using opt-in emails, co-registration with panel partners, e-newsletter campaigns, and online banner placements. All panelists must explicitly agree to be a panel member, register with the panel through a unique email address, and complete an in-depth demographic registration profile. In all countries, the primary method of data collection is through the Internet. However to ensure a representative sample in France, Germany, Italy, and Spain, particularly in the older population (>65), online recruitment was supported by computer assisted web interviews (CAWIs), where respondents were recruited by telephone and had the choice to complete the interview on the phone while the interviewer entered the responses online, or were e-mailed a link to the survey to complete on their own. The NHWS was approved by Essex Institutional Review Board (Lebanon, NJ, USA), and all respondents provided informed consent by selecting “I agree to participate” to the question “Do you voluntarily agree to participate in this study?”. Participants who completed the NHWS received compensation in the form of points, which could be redeemed for small prizes or entered into a drawing. All information was self-reported by respondents.

This study included combined data from the 2010, 2011 and 2013 5EU NHWS datasets (the 5EU NHWS was not fielded in 2012). The current study pooled together multiple years of data to increase the sample size of respondents providing care for an adult relative with schizophrenia. It is possible for a respondent to complete more than one survey over a several year period; only the most recent data for a given respondent was kept in these instances. All NHWS respondents were asked, “Are you currently caring for an adult relative with any of the following conditions?”, several response conditions were listed (*e.g.*, schizophrenia, Alzheimer’s disease, stroke, multiple sclerosis, epilepsy, and cancer). Data were analyzed for respondents who self-reported being a caregiver for an adult relative with schizophrenia and were compared to two groups i) respondents not providing care for an adult relative with any condition (non-caregivers) and ii) respondents who self-reported providing care for an adult with a condition other than schizophrenia (*e.g.*, Alzheimer’s disease, cancer, stroke, etc.) (other caregivers).

### Demographics and health characteristics

The following demographic and health characteristics were assessed: country, age (continuous), gender (male or female), marital status (married/living with partner *vs.* single/divorced/separated/widowed), education (college/university degree *vs.* less than college/university degree), household income [low (<€20,000/<£20,000), high (≥€50,000/≥£40,000), decline to answer *vs.* medium (€20,000 to <50,000/£20,000 to < £40,000)], employment status (currently employed (full-time, part-time or self-employed) *vs.* not currently employed), body mass index (BMI; overweight, obese, or decline to answer *vs.* underweight/normal weight) was calculated using height and weight information, smoking status (currently smoke, former smoker *vs.* never smoked), alcohol use (currently drink alcohol *vs.* do not drink alcohol), exercised vigorously for at least 20 min in the past 30 days (exercised at least once *vs.* not), and an adjusted Charlson comorbidity index (CCI) [17]. The CCI weights the presence of the following self-reported conditions and sums the result: HIV/AIDS, metastatic tumor, lymphoma, leukemia, any tumor, moderate/severe renal disease, hemiplegia, diabetes, mild liver disease, ulcer disease, connective tissue disease, chronic pulmonary disease, dementia, cerebrovascular disease, peripheral vascular disease, myocardial infarction, congestive heart failure, and diabetes with end organ damage. The original CCI predicts the likelihood of mortality. In the current study the CCI provides an estimate of comorbidity burden and the greater the total index score, the greater the comorbid burden on the individual.

### Dependent measures

HRQoL was assessed using the physical (PCS) and mental component summary (MCS) scores from the Short Form (SF)-12v2 (2010 NHWS) and SF-36v2 (2011 and 2013 NHWS), as well as the health utility measure (SF-6D) [18–20]. As a standard for the SF-36v2, PCS, and MCS scores are normed to the U.S. population (M = 50, SD = 10), with higher scores indicating greater HRQoL [8]. The health utility score is a preference-based single index measure for health using general population values, with higher scores indicating better health status. A difference greater than 3 in either PCS or MCS was regarded as a minimally important difference (MID) and a difference greater than 0.041 on health utilities was identified as a MID [21, 22].

Comorbidities were investigated through the presence of insomnia, narcolepsy, sleep difficulties (no definition of sleep difficulties was provide, sleep difficulties can overlap with insomnia or narcolepsy with self-reported data), experiencing pain (any kind), migraines, headaches, heartburn, anxiety, and depression with the self-reported measure “have you *experienced* the following in the past twelve months”. Symptoms of depression were also assessed using the Patient Health Questionnaire-9 (PHQ-9). The PHQ-9 is an instrument which incorporates the DSM-IV (Diagnostic and Statistical Manual of Mental Disorders, 4th Edition) depression diagnostic criteria which in turn measures the frequency of depression symptoms with each item scored on a 4-point response scale (not at all = 0 to nearly every day = 3) in the past two weeks [23]. The level of depression severity was assessed according to the following total scores: 0–4 = minimal, 5–9 = mild, 10–14 = moderate, 15–19 = moderately severe, 20–27 = severe. The PHQ-9 was only available for NHWS 2011 and 2013 5EU respondents (67.5 %). All respondents also self-reported if they were using a prescription medication for depression.

### Reaction to caregiving

The Caregiver Reaction Assessment (CRA) is a 24-item scale designed to measure the reactions of family members to caring for elderly relatives with a variety of illnesses and it consists of 5 subscales (*i.e.*, daily schedule, financial situation, relationships with others, physical health, and self-esteem) [24, 25]. The CRA corresponds to the theoretical constructs of the labor of caregiving by measuring the impact of “taking care” related to managing the environment, preparing for death, and knowing one’s strengths. Responses are measured on a 5-point Likert-type scale (strongly disagree to strongly agree). The CRA was only available for caregivers from the 2013 NHWS (*n* = 157). All individual items were dichotomized as agree/strongly agree *vs.* other.

### Statistical analyses

To analyze demographic, health status, and comorbidity differences between schizophrenia caregivers, and non-caregiver controls, and between schizophrenia caregivers, and other caregivers, bivariate analyses were performed. Chi-square tests were used with categorical variables; ANOVAs were used with continuous variables.

Covariates noted above (demographics and health characteristics) were entered into a single logistic regression model to predict providing care to an adult patient with schizophrenia *vs.* not providing care. Another separate logistic regression model was run to predict providing care to an adult patient with schizophrenia *vs.* those providing care for adults with a condition other than schizophrenia. Schizophrenia caregivers were matched to non-caregiver and other caregiver respondents on the propensity score using the “greedy” matching algorithm [26]. A 1:2 matching ratio was implemented, each schizophrenia caregiver was matched to two non-caregiver control respondents and separately to two caregivers of other conditions. Post-match, differences between these groups were re-examined to confirm sufficient matching. Also, the matching was constrained so that all matches were within each 5EU country.

Differences on HRQoL, and self-reported comorbidities were examined post-matching to quantify the burden of schizophrenia caregiving as a function of humanistic outcomes. Chi-square and ANOVA tests were used to test for statistical differences across i) those providing care for an adult relative with schizophrenia *vs.* those not providing care for an adult relative and ii) those providing care for an adult relative with schizophrenia *vs.* those providing care for an adult relative with a condition other than schizophrenia. Statistical significance was set at 2-tailed *p* <0.05.

## Results

A total of 398 schizophrenia caregivers, 158,989 non-caregivers controls and 14,341 caregivers of other conditions were identified *via* 5EU NHWS across 2010, 2011 and 2013. In this total sample of 173,728 adults across the 5EU, 25.4 % were in France, 25.3 % in Germany, 25.6 % in the UK, 14.0 % in Italy, and 9.6 % in Spain.

### Schizophrenia caregivers *vs.* non-caregivers

The average age of schizophrenia caregivers was 45.3 years (SD = 15.8 years), 59.6 % were female, 52.5 % were currently employed, and 14.8 % reported an income of ≥ €50,000/≥£40,000. Before matching, schizophrenia caregivers compared with non-caregivers, were more likely to be female (59.6 % *vs.* 51.4 %), less likely to be married/living with partner (57.4 % *vs.* 62.8 %), reported lower annual household income, were less likely to be employed (52.5 % *vs.* 57.7 %), more likely to currently smoke (36.7 % *vs.* 26.1 %), and reported greater comorbidity burden *via* the CCI, all *p* <0.05. No statistically significant differences on age, education level, BMI, alcohol use, and exercise behaviors were found between the two groups (see Table 1).

**Table 1:** Demographic and health characteristic information for schizophrenia caregivers and non-caregiver controls

	Total	Non-caregiver	Schizophrenia caregiver for adult relative	*P*-value
	(*N* = 159,387)	(*N* = 158,989)	(*N* = 398)	
Age (years)				*0.156*
Mean ± SD	46.44 ± 15.86	46.44 ± 15.86	45.31 ± 15.77	
Country				*<0.001*
France (%)	40399 (25.35 %)	40326 (25.36 %)	73 (18.34 %)	
Germany (%)	41176 (25.83 %)	41094 (25.85 %)	82 (20.60 %)	
Italy (%)	21708 (13.62 %)	21639 (13.61 %)	69 (17.34 %)	
Spain (%)	14936 (9.37 %)	14863 (9.35 %)	73 (18.34 %)	
UK (%)	41168 (25.83 %)	41067 (25.83 %)	101 (25.38 %)	
Gender				*0.001*
Female (%)	81915 (51.39 %)	81678 (51.37 %)	237 (59.55 %)	
Male (%)	77472 (48.61 %)	77311 (48.63 %)	161 (40.45 %)	
Marital status				*0.029*
Single (%)	59243 (37.17 %)	59074 (37.16 %)	169 (42.46 %)	
Married/living with partner (%)	100144 (62.83 %)	99915 (62.84 %)	229 (57.54 %)	
Education level				*0.051*
Less than college/university degree (%)	105151 (65.97 %)	104870 (65.96 %)	281 (70.60 %)	
College/university degree (%)	54236 (34.03 %)	54119 (34.04 %)	117 (29.40 %)	
Annual household income				*<0.001*
<€20,000/<£20,000 (%)	44431 (27.88 %)	44272 (27.85 %)	159 (39.95 %)	
€20,000 to < €50,000/£20,000 to < £40,000 (%)	67854 (42.57 %)	67693 (42.58 %)	161 (40.45 %)	
≥€50,000/≥£40,000 (%)	25151 (15.78 %)	25092 (15.78 %)	59 (14.82 %)	
Decline to answer (%)	21951 (13.77 %)	21932 (13.79 %)	19 (4.77 %)	
Employment status				*0.038*
Not currently employed (%)	67516 (42.36 %)	67327 (42.35 %)	189 (47.49 %)	
Employed (%)	91871 (57.64 %)	91662 (57.65 %)	209 (52.51 %)	
Body mass index				*0.996*
Underweight (%)	4483 (2.81 %)	4472 (2.81 %)	11 (2.76 %)	
Normal weight (%)	69560 (43.64 %)	69390 (43.64 %)	170 (42.71 %)	
Overweight (%)	52470 (32.92 %)	52338 (32.92 %)	132 (33.17 %)	
Obese (%)	28630 (17.96 %)	28556 (17.96 %)	74 (18.59 %)	
Decline to provide weight (%)	4244 (2.66 %)	4233 (2.66 %)	11 (2.76 %)	
Alcohol use				*0.281*
Do not drink alcohol (%)	34504 (21.65 %)	34409 (21.64 %)	95 (23.87 %)	
Drink alcohol (%)	124883 (78.35 %)	124580 (78.36 %)	303 (76.13 %)	
Smoking behavior				*<0.001*
Non-smoker (%)	65277 (40.96 %)	65165 (40.99 %)	112 (28.14 %)	
Former smoker (%)	52450 (32.91 %)	52310 (32.90 %)	140 (35.18 %)	
Current smoker (%)	41660 (26.14 %)	41514 (26.11 %)	146 (36.68 %)	
Exercise behavior				*0.505*
Do not exercise (%)	65460 (41.07 %)	65290 (41.07 %)	170 (42.71 %)	
Exercise at least once a month (%)	93927 (58.93 %)	93699 (58.93 %)	228 (57.29 %)	
Charlson comorbidity index				*<0.001*
Mean ± SD	0.28 ± 0.77	0.28 ± 0.77	0.61 ± 1.26	

After propensity matching, schizophrenia caregivers were more likely to report experiencing sleep difficulties (42.7 % *vs.* 28.5 %), insomnia (32.4 % *vs.* 18.5 %), pain (39.7 % *vs.* 30.4 %), headaches (48.0 % *vs.* 42.0 %), heartburn (31.7 % *vs.* 22.9 %), anxiety (37.9 % *vs.* 23.6 %), and depression (29.4 % *vs.* 19.4 %) in the past 12 months than non-caregivers, all *p* <0.05. Based on the PHQ-9, schizophrenia caregivers reported greater severity of depressive symptoms than non-caregivers (*p* <0.001). Schizophrenia caregivers were also more likely to currently be using a prescription medication to treat depression (17.6 % *vs.* 8.2 %, *p* <0.001) than non-caregiver controls. Schizophrenia caregivers reported significantly lower MCS (40.3 *vs.* 45.9), PCS (46.8 *vs.* 49.0), and health utility (0.64 *vs.* 0.71), compared with non-caregivers (all *p* <0.001) (see Table 2).

**Table 2:** Post-matched health outcome differences for schizophrenia caregivers vs. non-caregiver controls

	Total	Non-caregiver	Schizophrenia caregiver for adult relative	*P*-value
	(*N* = 1,194)	(*N* = 796)	(*N* = 398)	
PHQ-9 scale^a^				*<0.001*
Minimal (%)	386 (32.33 %)	307 (38.57 %)	79 (19.85 %)	
Mild (%)	221 (18.51 %)	137 (17.21 %)	84 (21.11 %)	
Moderate (%)	106 (8.88 %)	59 (7.41 %)	47 (11.81 %)	
Moderately Severe (%)	56 (4.69 %)	30 (3.77 %)	26 (6.53 %)	
Severe (%)	39 (3.27 %)	13 (1.63 %)	26 (6.53 %)	
Currently using a prescription medication for depression (%)	135 (11.31 %)	65 (8.17 %)	70 (17.59 %)	*<0.001*
Comorbidities experienced in the past 12 months				
Narcolepsy (%)	10 (0.84 %)	4 (0.50 %)	6 (1.51 %)	*0.072*
Insomnia (%)	276 (23.12 %)	147 (18.47 %)	129 (32.41 %)	*<0.001*
Sleep difficulties (%)	397 (33.25 %)	227 (28.52 %)	170 (42.71 %)	*<0.001*
Pain (%)	400 (33.50 %)	242 (30.40 %)	158 (39.70 %)	*0.001*
Anxiety (%)	339 (28.39 %)	188 (23.62 %)	151 (37.94 %)	*<0.001*
Depression (%)	271 (22.70 %)	154 (19.35 %)	117 (29.40 %)	*<0.001*
Heartburn (%)	308 (25.80 %)	182 (22.86 %)	126 (31.66 %)	*0.001*
Migraines (%)	284 (23.79 %)	178 (22.36 %)	106 (26.63 %)	*0.102*
Headaches (%)	525 (43.97 %)	334 (41.96 %)	191 (47.99 %)	*0.048*
Mental component summary				*<0.001*
Mean ± SD	44.01 ± 11.15	45.88 ± 10.87	40.25 ± 10.75	
Physical component summary				*<0.001*
Mean ± SD	48.23 ± 9.98	48.96 ± 9.79	46.77 ± 10.20	
Health utility				*<0.001*
Mean ± SD	0.69 ± 0.13	0.71 ± 0.13	0.64 ± 0.12	

^a^Only available for 2011 and 2013 NHWS respondents (546 non-caregivers, 262 schizophrenia caregivers); Patient Health Questionnaire-9 = PHQ-9

### Schizophrenia *vs.* other caregivers

Before propensity matching, schizophrenia caregivers compared with caregivers of other conditions, were younger (45.3 *vs.* 49.1 years), less likely to be married/living with a partner (57.4 % *vs.* 68.1 %), had lower annual household income, were more likely to currently smoke (36.7 % *vs.* 29.2 %), and reported greater comorbidity burden, all *p* <0.05. No statistically significant differences on gender, education level, employment status, BMI, alcohol use, and exercise behaviors were found between the two groups (see Table 3).

**Table 3:** Demographic and health characteristic information for schizophrenia caregivers and other caregivers

	Total	Caregiver for adult relative with a condition other than schizophrenia	Schizophrenia caregiver for adult relative	*P*-value
	(*N* = 14,739)	(*N* = 14,341)	(*N* = 398)	
Age (years)				*<0.001*
Mean ± SD	49.04 ± 14.96	49.14 ± 14.93	45.31 ± 15.77	
Country				*<0.001*
France (%)	3887 (26.37 %)	3814 (26.60 %)	73 (18.34 %)	
Germany (%)	2931 (19.89 %)	2849 (19.87 %)	82 (20.60 %)	
Italy (%)	2681 (18.19 %)	2612 (18.21 %)	69 (17.34 %)	
Spain (%)	1766 (11.98 %)	1693 (11.81 %)	73 (18.34 %)	
UK (%)	3474 (23.57 %)	3373 (23.52 %)	101 (25.38 %)	
Gender				*0.467*
Female (%)	8515 (57.77 %)	8278 (57.72 %)	237 (59.55 %)	
Male (%)	6224 (42.23 %)	6063 (42.28 %)	161 (40.45 %)	
Marital status				*<0.001*
Single (%)	4745 (32.19 %)	4576 (31.91 %)	169 (42.46 %)	
Married/living with partner (%)	9994 (67.81 %)	9765 (68.09 %)	229 (57.54 %)	
Education level				*0.121*
Less than college/university degree (%)	9875 (67.00 %)	9594 (66.90 %)	281 (70.60 %)	
College/university degree (%)	4864 (33.00 %)	4747 (33.10 %)	117 (29.40 %)	
Annual household income				*<0.001*
<€20,000/<£20,000 (%)	4299 (29.17 %)	4140 (28.87 %)	159 (39.95 %)	
€20,000 to < €50,000/£20,000 to < £40,000 (%)	6489 (44.03 %)	6328 (44.13 %)	161 (40.45 %)	
≥€50,000/≥£40,000 (%)	2200 (14.93 %)	2141 (14.93 %)	59 (14.82 %)	
Decline to answer (%)	1751 (11.88 %)	1732 (12.08 %)	19 (4.77 %)	
Employment status				*0.692*
Not currently employed (%)	6855 (46.51 %)	6666 (46.48 %)	189 (47.49 %)	
Employed (%)	7884 (53.49 %)	7675 (53.52 %)	209 (52.51 %)	
Body mass index				*0.663*
Underweight (%)	431 (2.92 %)	420 (2.93 %)	11 (2.76 %)	
Normal weight (%)	5901 (40.04 %)	5731 (39.96 %)	170 (42.71 %)	
Overweight (%)	4888 (33.16 %)	4756 (33.16 %)	132 (33.17 %)	
Obese (%)	3153 (21.39 %)	3079 (21.47 %)	74 (18.59 %)	
Decline to provide weight (%)	366 (2.48 %)	355 (2.48 %)	11 (2.76 %)	
Alcohol use				*0.424*
Do not drink (%)	3276 (22.23 %)	3181 (22.18 %)	95 (23.87 %)	
Drink alcohol (%)	11463 (77.77 %)	11160 (77.82 %)	303 (76.13 %)	
Smoking behavior				*<0.001*
Non-smoker (%)	5507 (37.36 %)	5395 (37.62 %)	112 (28.14 %)	
Former smoker (%)	4893 (33.20 %)	4753 (33.14 %)	140 (35.18 %)	
Current smoker (%)	4339 (29.44 %)	4193 (29.24 %)	146 (36.68 %)	
Exercise behavior				*0.613*
Do not exercise (%)	6114 (41.48 %)	5944 (41.45 %)	170 (42.71 %)	
Exercise at least once a month (%)	8625 (58.52 %)	8397 (58.55 %)	228 (57.29 %)	
Charlson comorbidity index				*<0.001*
Mean ± SD	0.44 ± 1.04	0.43 ± 1.04	0.61 ± 1.26	

After completing the propensity matching, schizophrenia caregivers were more likely to report experiencing sleep difficulties (42.7 % *vs.* 36.8 %), insomnia (32.4 % *vs.* 26.0 %), pain (39.7 % *vs.* 31.5 %), and anxiety (37.9 % *vs.* 29.8 %) than other caregivers, all *p* <0.05. Based on the PHQ-9, schizophrenia caregivers reported greater severity of depressive symptoms than other caregivers (p = 0.003). Schizophrenia caregivers were also more likely to be currently taking a prescription medication to treat depression (17.6 % *vs.* 11.4 %, p = 0.003), but only a marginal significant differences was found on schizophrenia caregivers experiencing depression in the past 12 months (p = 0.069) compared with caregivers of other conditions. Comparing schizophrenia caregivers and other caregivers, schizophrenia caregivers reported lower MCS (40.3 *vs.* 42.7, *p* <0.001), and health utilities (0.64 *vs.* 0.67, *p* <0.001). No statistically significant difference was found on PCS scores between schizophrenia caregivers and other caregivers (see Table 4).

**Table 4:** Post-matched health outcome differences for schizophrenia caregivers vs. other caregivers

	Total	Caregiver for adult relative with condition other than schizophrenia	Schizophrenia caregiver for adult relative	*P*-value
	(*N* = 1194)	(*N* = 796)	(*N* = 398)	
PHQ-9 scale^a^				*0.003*
Minimal (%)	302 (25.29 %)	223 (28.02 %)	79 (19.85 %)	
Mild (%)	223 (18.68 %)	139 (17.46 %)	84 (21.11 %)	
Moderate (%)	151 (12.65 %)	104 (13.07 %)	47 (11.81 %)	
Moderately Severe (%)	74 (6.20 %)	48 (6.03 %)	26 (6.53 %)	
Severe (%)	52 (4.36 %)	26 (3.27 %)	26 (6.53 %)	
Currently using a prescription medication for depression (%)	161 (13.48 %)	91 (11.43 %)	70 (17.59 %)	*0.003*
Comorbidities experienced in the past 12 months				
Narcolepsy (%)	11 (0.92 %)	5 (0.63 %)	6 (1.51 %)	*0.134*
Insomnia (%)	336 (28.14 %)	207 (26.01 %)	129 (32.41 %)	*0.020*
Sleep difficulties (%)	463 (38.78 %)	293 (36.81 %)	170 (42.71 %)	*0.048*
Pain (%)	409 (34.25 %)	251 (31.53 %)	158 (39.70 %)	*0.005*
Anxiety (%)	388 (32.50 %)	237 (29.77 %)	151 (37.94 %)	*0.005*
Depression (%)	312 (26.13 %)	195 (24.50 %)	117 (29.40 %)	*0.069*
Heartburn (%)	348 (29.15 %)	222 (27.89 %)	126 (31.66 %)	*0.177*
Migraines (%)	331 (27.72 %)	225 (28.27 %)	106 (26.63 %)	*0.552*
Headaches (%)	557 (46.65 %)	366 (45.98 %)	191 (47.99 %)	*0.512*
Mental component summary				*<0.001*
Mean ± SD	41.85 ± 10.72	42.65 ± 10.62	40.25 ± 10.75	
Physical component summary				*0.315*
Mean ± SD	47.19 ± 10.10	47.40 ± 10.05	46.77 ± 10.20	
Health utility				*<0.001*
Mean ± SD	0.66 ± 0.13	0.67 ± 0.13	0.64 ± 0.12	

^a^Only available for 2011 and 2013 NHWS respondents (540 other caregivers, 262 schizophrenia caregivers); Patient Health Questionnaire-9 = PHQ-9

### Schizophrenia caregivers: reaction to caregiving

**Fig. 1:**
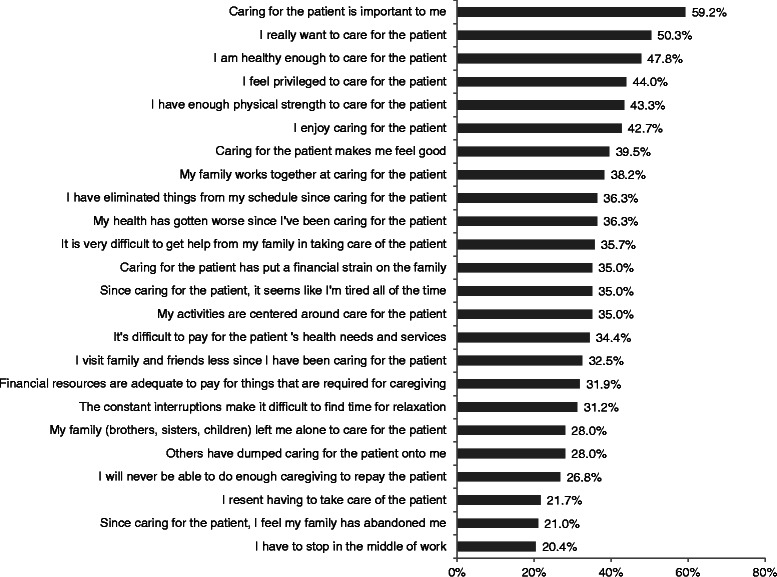
Caregiver reactions assessment % agree/strong agree for schizophrenia caregivers (2013 only *n* = 157; 39.4 % of schizophrenia caregivers)

Notably, when asked about their caregiving experience, the top 7 items (in terms of frequency) were positive about the experience. The majority of caregivers responded that caring for the patient was important to them (59.2 %) and that they wanted to care for the patient (50.3 %). A substantial proportion of caregivers responded that they were healthy enough to care for the patient (47.8 %), feeling privileged to care for the patient (44.0 %), having enough physical strength to care for the patient (43.3 %), enjoyed caring for the patient (42.7 %), and that caring for the patient makes them feel good (39.5 %) (see Fig. 1).

However, a substantial proportion of caregivers indicated that the caregiving experience was burdensome. In particular, caregivers indicated that caregiving had disrupted their schedules (36.3 %), that their health had become worse since starting caregiving (36.3 %), feeling tired all the time since starting care for the patient (35.0 %), not visiting friends and family as much as before (32.5 %), having difficulty relaxing because of constant disruptions (31.2 %), and having to stop in the middle of work (20.4 %).

A substantial proportion of caregivers indicated inadequate support caring for the patient. In particular, caregivers responded that they had financial difficulty with the patient’s needs and services (34.4 %), that the care had put a financial strain on their family (35.0 %), that it is difficult to get help from their family (35.7 %), that their family left them alone to take care of the patient (28.0 %), their family “dumped” caring for the patient on them (28.0 %), and that their family abandoned them since starting care (21.0 %).

## Discussion

In general, informal schizophrenia caregivers exhibited poorer health-related outcomes than non-caregiver controls. After matching schizophrenia caregivers with non-caregivers with similar demographic and health characteristics, a substantially greater proportion of caregivers reported experiencing the following symptoms and conditions: sleep difficulties, insomnia, pain, headaches, heartburn, anxiety, and depression, all *p* <0.05. Schizophrenia caregivers also reported lower HRQoL and health utility compared with non-caregiver controls, all *p* <0.05. Indeed, the mean differences between schizophrenia caregivers and non-caregiver controls were larger than the MID for mental HRQoL and health utility.

Caregivers of schizophrenia patients and caregivers of patients with conditions other than schizophrenia reported similar poor health-related outcomes, although some differences emerged. After matching schizophrenia caregivers with caregivers of patients with conditions other than schizophrenia but with similar demographic and health characteristics, a substantially greater proportion of schizophrenia caregivers reported the following symptoms: sleep difficulties, insomnia, and anxiety, all *p* <0.05. Moreover, a substantially greater proportion of schizophrenia caregivers reported currently taking prescription medication for depression and a greater level of depression severity. Schizophrenia caregivers exhibited significantly lower mean mental HRQoL and health utility scores compared with caregivers of patients with other conditions, though these differences did not exceed our pre-defined threshold of meaningfully important differences, all *p* <0.05.

A prior review of published research of schizophrenia caregiver burden found that, overall, this population experiences deteriorated health, with stress problems, anxiety and depression [27]. The current study corroborated these findings, as informal schizophrenia caregivers reported higher levels of these health issues relative to non-caregivers and caregivers of conditions other than schizophrenia. Zendijidjian *et al.* (2012) found that caregivers of patients with affective disorders scored significantly lower on all SF-36 domains than caregivers of schizophrenia patients [15]. The current study, however, found significant differences on the MCS, but not the PCS when comparing schizophrenia caregivers and caregivers of other conditions. These differences could be due to the broader criteria provided for caregivers of other conditions in the current study. Papastavrou (2012), comparing schizophrenia, Alzheimer’s and cancer caregivers, on the other hand, found that caregivers of cancer patients experienced the highest levels of depression, while Alzheimer’s caregivers experienced the highest levels of overall burden (*p* <0.001) [16]. Unlike previous studies of schizophrenia caregivers, the current study employed a representative sample of schizophrenia caregivers, directly comparing HRQoL and comorbidities for schizophrenia caregivers with non-caregiver controls, and schizophrenia caregivers with other caregivers. Because of this, making direct comparisons with prior studies is limited. However, a prior study using 2010 and 2011 5EU NHWS reports higher MCS, PCS and health utility scores for cancer caregivers than the current studies schizophrenia caregivers [28], suggesting potentially poorer HRQoL for schizophrenia caregivers than caregivers of cancer patients. Therefore, overall, given previous literature and the current study results, the health status of schizophrenia caregivers were found to be comparable if not worse than caregivers of other conditions.

The number of published studies elucidating the negative aspects of caregiving for patients with chronic illness substantially outweighs the number of studies elucidating the positive aspects [13]. Indeed, the current study is unique in reporting the results of the CRA in a schizophrenia caregiver population, corroborating prior research suggesting that even though caregiving is often burdensome, caregivers are able to have positive experiences. The current study follows European region WHO Mental Health Plan guidelines in that its aim, in part, was to assess the ability of caregivers to cope with their caregiving experience [14, 17]. Notably, when asked about their caregiving experience, the most frequent responses were positive. The majority of caregivers indicated they wanted to care for the patient and that caring for the patient was important to them. However, a substantial proportion of informal caregivers indicated that caregiving was an objective burden, in the form of disrupting their daily routines and work, and in limiting their social life. The responses suggested that caregiving was also subjectively burdensome as caregivers indicated their health had become worse, feeling tired all the time, and having difficulty relaxing because of constant interruptions since starting care. A substantial proportion of caregivers indicated that they lacked social and financial support, feeling that their family had left them sole responsibility with caring for the patient, and being isolated from the family and their friends. Prior studies which utilized the CRA corroborated these findings with caregiver responses being relatively low for questions that indicated caregiver resentment and high for questions regarding caregiver self-esteem, over a variety of caregiver populations [29–33]. Also in agreement with the current findings, the studies consistently found that caregivers experience high levels of financial burden, often lack social support from family and formal sources, and that caregiving responsibilities interfering with caregivers’ schedules is a prominent source of burden. Direct comparisons between these studies, however, are not feasible because of inconsistent statistical reporting. Moreover, the CRA lacks a composite index summarizing total caregiving burden making comparisons of relative burden between caregiving populations difficult.

Together, these results suggest that while informal caregivers want to care for their patients and feel privileged to provide care, caregiving has an impact on caregivers both objectively and subjectively. While the current study is limited in being able to elucidate the causal direction because of its cross-sectional nature, it is likely that the observed differences in HRQoL and comorbidities are caused by the burden of caregiving. Alternatively, it is possible that individuals with lower HRQoL and greater comorbidities self-select or are put into caregiving roles more frequently. However, the symptoms and conditions that were assessed (sleep difficulties, insomnia, pain, headaches, heartburn, anxiety, depression, etc.) are all associated with chronic stress, and it seems more likely that these would be caused by caregiving burden than being predispositions for becoming a caregiver. Indeed, 36 % of caregivers indicated that their health had become worse since starting caregiving.

Further, providing care for an adult relative with schizophrenia is important to caregivers, but the results suggest caregivers may benefit from additional financial and social support, and coping strategy, programs (as described in the European region WHO Mental Health Plan) as a substantial number of caregivers indicated inadequate resources to fulfill their care. As prior research suggests that patient symptomatology has a meaningful impact on the severity of caregiver burden [9]. Also, better treatment options for patients with schizophrenia or more-adequately treated patients would have the added benefit of also alleviating caregiving burden [1]. Indeed, prior research suggests that, on a population-wide scale, this would amount to substantial humanistic and economic benefit to society because of the “spill-over” effect that chronic conditions have on patients’ families [1, 13].

### Limitations

Although a representative sample of the 5EU, this sample may not have been representative of schizophrenia caregivers, caregivers of conditions other than schizophrenia, and non-caregiver controls. These analyses included many covariates in the models, but other relevant covariates may not have been included, such as the caregiver’s type of employment and length of time providing care for the patient with schizophrenia. Also, data on caregiver relationship, patient symptoms, and patient treatments were not collected, which could also have an impact of the amount of caregiver burden. All responses were self-reported and may reflect recall biases and other forms of measurement error.

Because the majority of respondents were surveyed *via* the internet, it is possible that the sample included younger caregivers, who are more likely to be educated on how to use the internet, and older caregivers who are more educated, and thus potentially healthier, than the caregiver population at large. However, consideration was taken to match caregivers to controls with similar sociodemographic characteristics, and therefore mean differences derived from these analyses were less likely biased due to oversampling healthier caregivers.

Although consideration have been given to explore alternative explanations for the observed mean differences in health outcomes, it is possible that unmeasured variables may have confounded the analyses. Moreover, schizophrenia diagnoses and caregiver status were not confirmed, and it is possible that survey respondents may have misclassified themselves as caregivers of schizophrenia patients. The use of a cross-sectional design precludes the ability to draw causal inferences from the data.

## Conclusions

In conclusion, informal schizophrenia caregivers reported worse HRQoL, health utilities, and experienced a greater number of comorbidities than non-caregivers and caregivers of other conditions. However, providing care for an adult relative with schizophrenia is important to caregivers and they feel privileged to provide care, but caregivers needed more resources to provide adequate care. Providing informal schizophrenia caregivers with support services (*e.g.*, financial support, social support, and coping strategy programs) to help better manage patients may improve their overall health status.
